# Synthetic Thyroid Hormone Receptor-β Agonists Promote Oligodendrocyte Precursor Cell Differentiation in the Presence of Inflammatory Challenges

**DOI:** 10.3390/ph16091207

**Published:** 2023-08-25

**Authors:** Vito Antonio Baldassarro, Corinne Quadalti, Massimiliano Runfola, Clementina Manera, Simona Rapposelli, Laura Calzà

**Affiliations:** 1 Department of Veterinary Medical Science (DIMEVET), University of Bologna, 40064 Bologna, Italy; vito.baldassarro2@unibo.it; 2 Department of Pharmacy and Biotechnology (FaBit), University of Bologna, 40126 Bologna, Italy; corinne.quadalti2@unibo.it; 3 Department of Pharmacy, University of Pisa, 56126 Pisa, Italy; massimiliano.runfola@pharm.ox.ac.uk (M.R.); clementina.manera@unipi.it (C.M.); simona.rapposelli@unipi.it (S.R.); 4 IRET Foundation, Ozzano Emilia, 40064 Bologna, Italy

**Keywords:** thyroid hormone receptors, oligodendrocyte precursor cells, TG68, thyromimetics, inflammation

## Abstract

Oligodendrocytes and their precursors are the cells responsible for developmental myelination and myelin repair during adulthood. Their differentiation and maturation processes are regulated by a complex molecular machinery driven mainly by triiodothyronine (T3), the genomic active form of thyroid hormone, which binds to thyroid hormone receptors (TRs), regulating the expression of target genes. Different molecular tools have been developed to mimic T3 action in an attempt to overcome the myelin repair deficit that underlies various central nervous system pathologies. In this study, we used a well-established in vitro model of neural stem cell-derived oligodendrocyte precursor cells (OPCs) to test the effects of two compounds: the TRβ1 ligand IS25 and its pro-drug TG68. We showed that treatment with TG68 induces OPC differentiation/maturation as well as both the natural ligand and the best-known TRβ1 synthetic ligand, GC-1. We then described that, unlike T3, TG68 can fully overcome the cytokine-mediated oligodendrocyte differentiation block. In conclusion, we showed the ability of a new synthetic compound to stimulate OPC differentiation and overcome inflammation-mediated pathological conditions. Further studies will clarify whether the compound acts as a pro-drug to produce the TRβ1 ligand IS25 or if its action is mediated by secondary mechanisms such as AMPK activation.

## 1. Introduction

Oligodendrocytes (OLs) are the cells responsible for myelination of the central nervous system (CNS) during development and for myelin turnover and repair in adulthood. They differentiate from OPCs through a strictly regulated molecular machinery that controls the equilibrium between the proliferative and differentiation states in functional OLs. The final maturation phase leads to the wrapping of lipid-enriched cell body protrusions around the unmyelinated axons to generate myelin sheets [1,2].

A similar process takes place when myelin is damaged, with new OPCs being generated and recruited to repair the lesion. This myelin repair process, known as remyelination, is the only CNS repair capability with the potential for complete functional and anatomical recovery [1,3,4]. In some demyelinating diseases, however, such as multiple sclerosis (MS) or spinal cord injury (SCI), this process becomes progressively less efficient, leading to a pathological feature known as remyelination failure [5,6]. In conditions characterized by persistent inflammation, OPCs retain their capability to proliferate and migrate to demyelinated axons but lose their ability to differentiate into myelinating OLs. This OPC differentiation block is therefore regarded as one of the main causes of remyelination failure in severe inflammatory conditions [7,8], and corroborates the hypothesis that a successful remyelination process depends on OPC differentiation [9,10]. The vulnerability of the OPC and the interference with its differentiation underlying the physiological turnover of mature oligodendrocytes are also regarded as main players not only in MS but in several neurological disorders [11].

OPC differentiation is regulated by a complex machinery involving several cellular and molecular players [12], including extracellular matrix components, neurotransmitters, growth factors, hormones, axonal signals, cytokines, and other soluble factors [13,14], playing different roles depending on the differentiation stage [15]. Of these, thyroid hormones (THs) are recognized as the main drivers of cell cycle exit and differentiation promotion via a combination of genomic and non-genomic mechanisms [16,17,18]. The genomic action is mediated by triiodothyronine (T3) [19], which acts as a ligand of the TH receptors (TRα, TRβ1, and TRβ2). TRs are transcription factors belonging to the superfamily of nuclear receptors (NRs), with the ability to activate/inactivate the expression of target genes through a molecular machinery that includes ligands, receptor dimerization with other NRs, and the recruitment of specific co-activator/co-suppressors [13,20,21].

Inflammatory cytokines severely alter T3 tissue molecular signaling, leading to tissue/cell hypothyroidism. In fact, our and other groups have demonstrated that OPC differentiation/maturation impairment in experimental allergic encephalomyelitis (EAE), an experimental model for MS, is accompanied by an increase in the T3-inactivating enzyme deiodinases 3 (D3) and dysregulation of TR mRNA expression [22,23,24]. We have also demonstrated that inflammation-induced tissue hypothyroidism can be partially overcome in vivo by exogenous T3 administration, observing that OPC differentiation and maturation are restored in T3-treated animals, resulting in improved myelin repair in EAE [22] and SCI [25]. This result has been confirmed by in vitro experiments in which we have demonstrated that inhibition of inflammation-induced D3 hyperactivity restores OPC differentiation [26,27].

Unfortunately, systemic T3 administration leads to severe side effects (thyrotoxicosis), prompting the development of synthetic thyromimetic ligands for TRα and/or TRβ over the last two decades. Based on the hypothesis that TRβ plays a key role in OPC differentiation [28], in this study we investigate the differentiation potential of two halogen-free thyromimetics, TG68 and IS25, recently identified as novel TRβ agonists [29]. Given the critical role of the T3/thyroid hormone receptor (T3/TR) axis in non-alcoholic steatohepatitis (NASH) and hepatocellular carcinoma (HCC), we recently investigated both compounds for the treatment of these pathological conditions, confirming their beneficial effects in pre-clinical studies on animals [30,31]. More importantly, both compounds also showed a strong hepatomitogenic effect in rats with no detectable systemic side effects, suggesting their therapeutic potential for various human diseases, including conditions associated with impaired regenerative capacity [32].

Based on these encouraging results, this study investigated the therapeutic potential of TG68 and IS25 in the remyelination process. We used a validated cell platform coupled with high content screening (HCS) imaging techniques based on OPCs derived from neural stem cells (NSCs), thus recapitulating the entire differentiation process [33].

## 2. Results

### 2.1. The Synthetic TRβ Ligand TG68 Efficiently Induces OPC Differentiation from NSCs

We tested the TRβ ligands TG68 and IS25 on OPCs generated from fetal NSCs. When seeded in an undifferentiated state, the culture consisted of approximately 70% OPCs and 30% astrocytes. After three days as an adhesion culture, OPC differentiation was induced by removing the OPC medium containing bFGF and PDGF-AA and replacing it with the differentiation medium containing CNTF, NAC, and T3, the main trigger of OPC differentiation, which occurs at 12 days. Blocking the culture at different time points allows investigation of how the molecular signature evolves over the 12 day differentiation period (Supplementary Figure S1) [34].

**Figure 1:**
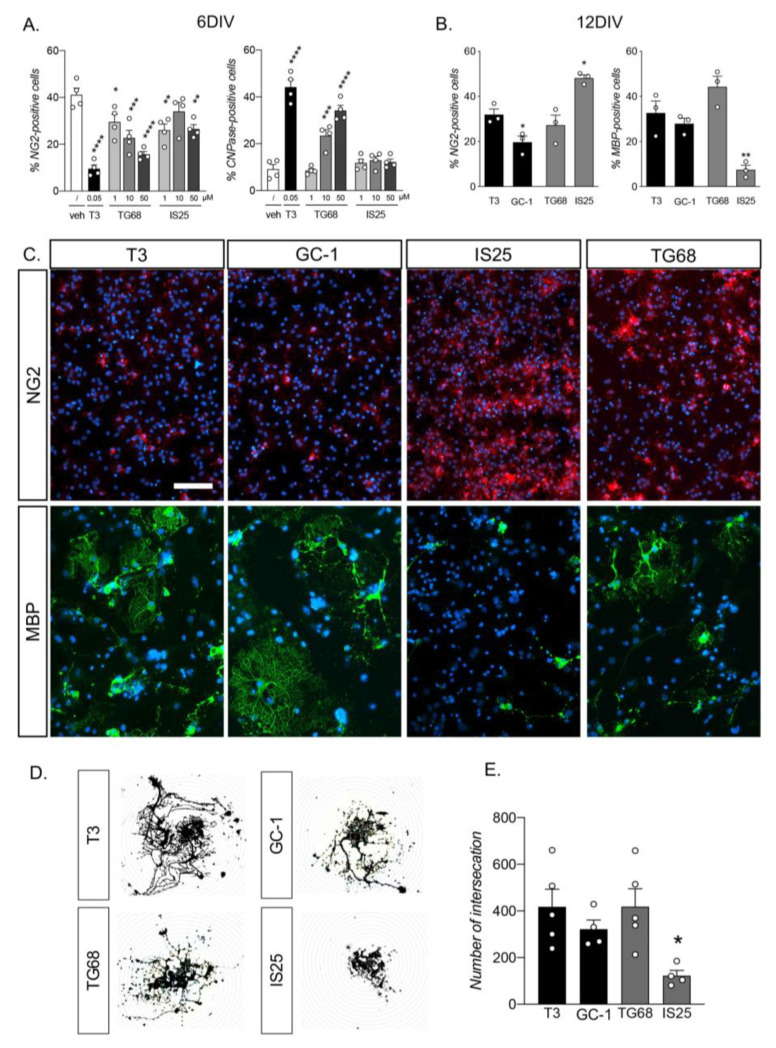
Analysis of the OPC differentiation induced by synthetic TRβ ligands compared to the physiological T3-mediated stimulus. (**A**,**B**) Graphs show the percentage of NG2- and CNPase-positive cells at six (**A**) and twelve (**B**) DIV following exposure to the different TR ligands. The 10 µM concentration was used at 12 DIVs. (**C**) Representative HCS images of cultures exposed to T3, GC-1, IS25, or TG68 after 12 DIVs of treatment, stained to detect OPCs (NG2) and late-mature OLs (MBP). Epifluorescence images of MBP-positive cells are included for each group. Bars: 150 µm. (**D**) Representative images of the Sholl analysis performed on MBP-positive cells. (**E**) The graph shows the Sholl analysis results expressed as the number of intersections. Statistical analysis. Bars represent the mean + SEM; *n* = 4 (**A**,**E**), *n* = 3 (**B**) independent replicates, indicated as circles inside each histogram. One-way ANOVA followed by Dunnett’s post-test on vehicle-treated cultures (**A**) or T3-treated cells (**B**,**E**). Asterisks represent differences between the indicated group and the control group (* *p* < 0.05; ** *p* < 0.01; *** *p* < 0.001; **** *p* < 0.0001).

The experiments were carried out using three different ligand dosages (1, 10, and 50 µM), T3 as a reference compound, and vehicle alone. The lineage progression was analyzed by automatic counting of NG2- (undifferentiated precursors), CNPase- (myelinating OLs), and MBP-positive cells at six and twelve DIVs. Six days of exposure to the physiological T3 and to the two synthetic TRβ ligands significantly reduced the percentage of OPCs in the culture compared to the vehicle (one-way ANOVA, F(7,24) = 13.45, *p* < 0.0001). As expected, T3 exposure induced a significant reduction of NG2-IR OPCs compared to vehicle-treated cultures (Dunnett’s post-test, *p* < 0.0001). All three tested TG68 doses also induced a reduction in the precursor cells (1 µM, *p* = 0.0317; 10 µM, *p* = 0.0005; and 50 µM, *p* < 0.0001), as did the doses of IS25, albeit less effectively, with a significant reduction for the 1 µM (*p* = 0.0036) and 50 µM (*p* = 0.0050) doses only. Analysis of the mature CNPase-IR OL population also showed an effect mediated by the TR ligand exposure (one-way ANOVA, F(7,24) = 43.17, *p* < 0.0001), but only for the T3 positive control (Dunnett’s post-test, *p* < 0.0001) and the two higher doses of TG68 (10 µM, *p* = 0.0003; 50 µM, *p* < 0.0001) (Figure 1A). The HCS-based assay included analysis of the nuclear morphology via quantification of the condensed nuclei (high Hoechst intensity and small area) as a sign of cell death [33], finding no differences among the experimental groups.

From the six DIV experiments, TG68 emerged as the most promising synthetic TRβ ligand; therefore, we chose the lowest effective dose (10 µM) to perform a longer exposure, reaching 12 DIVs of differentiation induction. We compared the effect of the two test drugs to the T3 physiological differentiation stimulus and to GC-1, the gold standard synthetic TRβ ligand [35], calculating and reporting the percentage of NG2-positive (undifferentiated precursors) and MBP-positive cells (mature-myelinating OLs) (Figure 1B). The synthetic ligands produced a different effect on the physiological induction by T3 (one-way ANOVA, F(3,8) = 16.34, *p* = 0.0009), with a higher efficacy of GC-1 (Dunnett’s post-test, *p* = 0.0478) and a very low efficacy of IS25 (*p* = 0.0124), while TG68 showed no differences (*p* = 0.5731) (Figure 1E). We also detected differences between groups in terms of OLs at a late stage of maturation (one-way ANOVA, F(3,8) = 15.56, *p* = 0.0011). However, only IS25 showed a very low differentiation induction efficiency compared to T3 (Dunnett’s post-test, *p* = 0.0047), while GC-1 and TG68 showed no differences from the physiological stimulus (GC-1, *p* = 0.7307; TG68, *p* = 0.1562) (Figure 1B).

Representative images acquired with a standard epifluorescence microscope are given in Figure 1C, while HCS-derived acquisitions are included in Supplementary Materials (Supplementary Figure S2), clearly indicating the different maturation stages of OLs exposed and unexposed to the different TRβ ligands. To objectively evaluate OL maturation, we performed the Sholl analysis on the MBP-labeled cells. This analysis quantifies the complexity of the spider-net-shaped cell membrane of the mature OLs, which is directly proportional to the level of maturation (Figure 1D). Treatment with the different TR ligands evoked different effects on oligodendrocyte maturation (one-way ANOVA, F(3,14) = 4.566, *p* = 0.0197): while cultures exposed to GC-1 and TG68 showed no differences in OL maturation compared to the physiological T3 ligand, the OLs treated with IS25 showed a very low level of maturation, expressed as the number of intersections with the Sholl analysis (Dunnett’s post-test, *p* = 0.0153) (Figure 1E).

Since the spontaneous mixed culture includes the presence of astrocytes, we also quantified the GFAP-positive population. Results are shown in Supplementary Materials (Supplementary Figure S3), showing a proportional change in astrocyte percentage according to the change in the oligodendroglial population, while no changes in cell morphology have been detected.

### 2.2. TRβ Downstream Signaling by TG68 and Other TR Ligands

**Figure 2:**
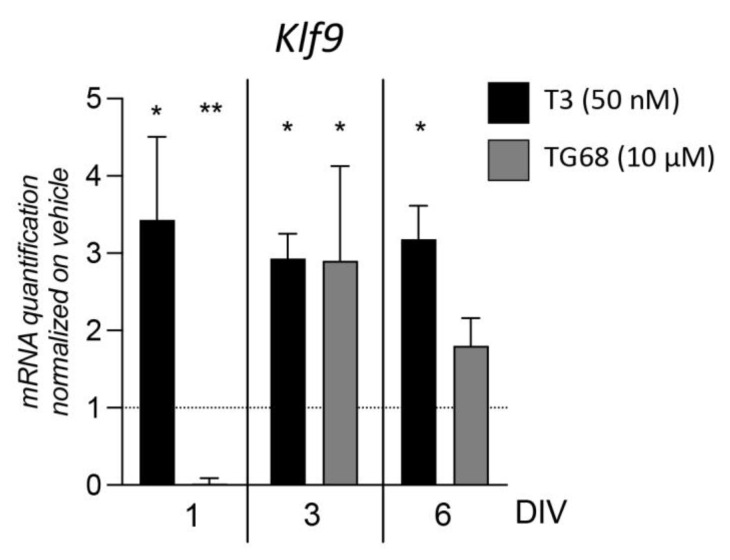
*Klf9* gene expression regulation by TRβ ligands. The graph shows the quantification of *Klf9* gene expression normalized to the vehicle-treated group for each time point (vehicle = one; horizontal dotted line). Statistical analysis. Bars represent the mean + SEM; *n* = three independent replicates. One-way ANOVA followed by Dunnett’s post-test on vehicle-treated cells (horizontal dotted line). Asterisks represent differences between the T3- or TG68-exposed groups and the vehicle-treated group (* *p* < 0.05; ** *p* < 0.01).

Having observed the efficiency of the new synthetic ligand TG68 at stimulating OPC differentiation and OL maturation in a manner similar to the physiological stimulus, we then investigated whether the TR-mediated activation of the differentiation process follows the same path between TG68 and T3. To this end, we analyzed the gene expression of *Klf9*, a specific ligand-dependent TRβ target that plays a key role in T3-mediated OPC differentiation [36], quantifying the *Klf9* expression in cultures treated with vehicle, T3, or TG68 at three different time points (one, three, and six DIVs) throughout the differentiation phase (Figure 2). *Klf9* expression was modified by TRβ ligand exposure (one-way ANOVA, F(2,10) = 13.94, *p* = 0.0013) twenty-four hours after differentiation induction (1 DIV), with a huge increase due to T3 action (Dunnett’s post-test, *p* = 0.0345), while TG68 caused a reduction (*p* = 0.0025). Expression was also modified at three DIVs (one-way ANOVA, F(2,10) = 5.451, *p* = 0.0251): T3-treated cells maintained a higher level of expression compared to vehicle-treated cultures (Dunnett’s post-test, *p* = 0.0437), while TG68-treated cells reached the same level of expression with a 3-fold increase (*p* = 0.0457). Although expression was also modified by the molecules at six DIVs (one-way ANOVA, F(2,10) = 5.099, *p* = 0.0298), while the *Klf9* genes showed the same level of upregulation in T3-treated cells (Dunnett’s post-test, *p* = 0.0207), expression in the TG68-treated cultures returned to the level of the vehicle-treated cells.

### 2.3. The Synthetic TRβ Ligand TG68 Reverts Cytokine-Induced OPC Differentiation Impairment More Efficiently Than T3

**Figure 3:**
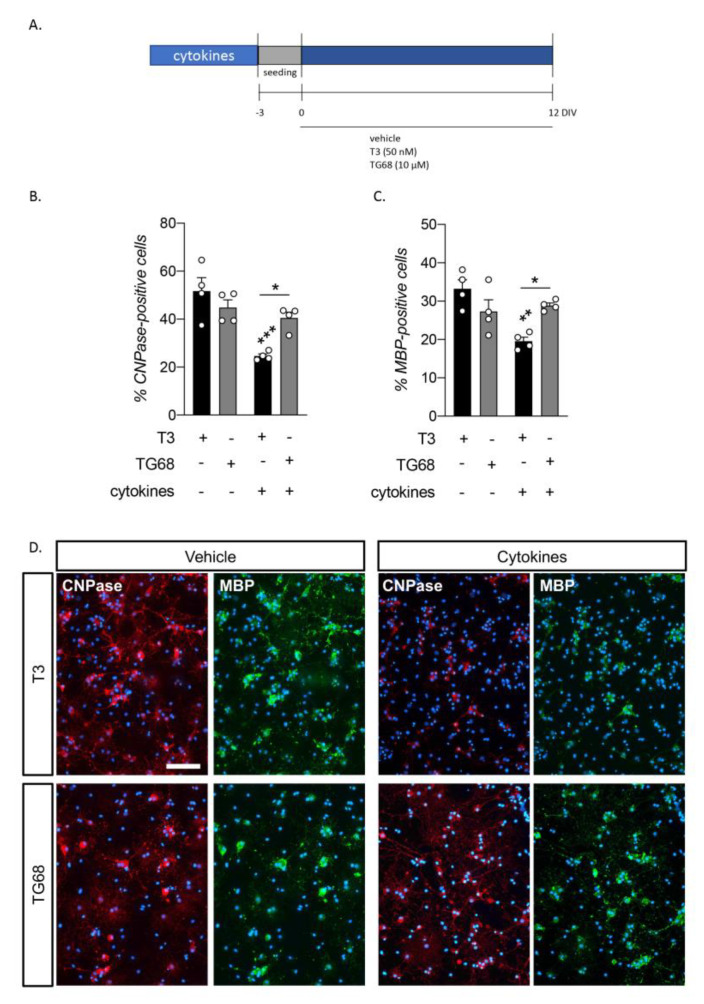
Rescue of cytokine-induced OPC differentiation by TRβ ligands. (**A**) Experimental protocol. OPCs were obtained from NSCs. During the second phase of expansion (oligosphere phase), cells were exposed to the cytokine mix for seven DIVs. Oligospheres were then dissociated and seeded as a single-cell suspension. After three DIVs (days in vitro), cells were exposed to the different TRβ ligands. The differentiation state of the cultures was assessed by analyzing the percentage of CNPase-positive (early-mature OLs) and MBP-positive (late-mature OLs) cells 12 DIV after differentiation induction. (**B**,**C**) Graphs show the percentage of CNPase- (**B**) and MBP- (**C**) positive cells after 12 DIVs of exposure to the different molecules. (**D**) Representative HCS images of cultures exposed and non-exposed to cytokines and treated with T3 or TG68 after 12 DIVs, stained to detect early-mature OLs (CNPase) and late-mature OLs (MBP). Bar: 150 µm. Statistical analysis. Bars represent the mean + SEM; *n* = 4 independent replicates, indicated as circles inside each histogram. One-way ANOVA followed by Tukey’s post-test. Asterisks represent differences between the indicated groups and the T3 control group, or between the groups indicated by a horizontal line (* *p* < 0.05; ** *p* < 0.01; *** *p* < 0.001).

Following the results observed in OPC differentiation and OL maturation, we chose TG68 as a candidate molecule to be tested in the in vitro model of inflammation-mediated OPC differentiation block, one of the main conditions leading to remyelination failure in MS and SCI. We used the NSC-derived OPC cultures combined with exposure to a cytokine mix, using cytokines selected directly from the EAE model and with proven efficacy at recapitulating the OPC differentiation block in vitro [26,27]. Following the standard protocol, cultures were exposed to the cytokine mix as 3D spheroids (oligospheres) during the expansion phase, and cells were treated with T3 or TG68 three days after seeding to induce differentiation. We then analyzed the percentage of CNPase and MBP-IR cells as markers for early- and late-OL differentiation (Figure 3A).

Our results showed the CNPase-IR cell percentage to be modified by the treatments (one-way ANOVA, F(3,12) = 11.01, *p* = 0.0009). Cytokine exposure generated a reduction in the number of early-mature OLs in the cultures not rescued by T3 (Tukey’s post-test, *p* = 0.0006), as was expected, while TG68 rescued the cytokine differentiation block almost completely, with no difference in the percentage of CNPase-positive cells compared to cultures unexposed to cytokines (*p* = 0.0316) (Figure 3B).

A similar trend is observed in fully differentiated MBP-IR cells (one-way ANOVA, F(3,12) = 7.939, *p* = 0.0035) following the same trend, with a lower percentage of MBP-positive cells in cytokine-exposed cultures treated with T3 (Tukey’s post-test, *p* = 0.0022), while no influence of the cytokine mix was observed in TG68-treated cells, which reached a higher maturation level compared to T3 (*p* = 0.0307) (Figure 3C).

Representative images acquired with a standard epifluorescence microscope are given in Figure 3D, while HCS-derived acquisitions are included in Supplementary Materials (Supplementary Figure S4).

## 3. Discussion

The genomic action of the T3 via TR activation is the main mechanism driving the OPC throughout the differentiation process, which generates mature OLs [37]. Since the alteration of this molecule at the intracellular level is regarded as the main cause of the OPC differentiation block, mediating the remyelination failure process [38,39,40], synthetic TR ligands have been proposed as therapeutic molecules.

In this study, we describe the action of TG68 and IS25, two novel synthetic TRβ ligands that induce OPC differentiation and maturation in vitro. Observing TG68 to be the most efficient molecule, we then tested its therapeutic potential in an in vitro model of cytokine-mediated OPC differentiation block.

### 3.1. TRβ1 Agonists and OPC Differentiation

The endogenous TR ligand T3 exerts a wide range of effects in different cell types, due in part to the cell-specific expression of TR isoforms. TRα1, for example, is mainly associated with cardiovascular functions [41], while TRβ1 is linked to obesity and metabolic disorders [42,43]. Despite extensive research into the selectivity of these ligands for the respective TRs [44], the molecular mechanisms supporting the downstream systemic effects remain unclear. For example, the TRβ-selective ligand GC-1 (commercially known as Sobetirome and QRX-431), one of the best characterized TRβ1-selective TH analogs, forms different complexes (including some with TRα) in several unstable conformational states [42].

OPCs express both TRα and TRβ1 [45], the latter believed to be the isoform chiefly responsible for T3-mediated differentiation induction [28,46,47,48]; indeed, we have previously described that TRβ is up-regulated over 20-fold following T3 exposure [34], and the TRβ-specific synthetic ligand GC-1 has been shown to exert a strong pro-differentiating effect [28]. In this study, we used our well-characterized in vitro system based on NSC-derived OPC cultures [33] to test two novel compounds, IS25 and TG68, designed as TRβ ligands. Compared to the natural ligand (T3), TG68 (but not IS25) was able to induce differentiation toward the final stage of maturation, as described by the MBP expression and induction of the spider-net morphology. TRβ activation by TG68 was also confirmed at the genomic level by the gene expression induction of *Klf9*, a direct target of the nuclear receptor. It is noteworthy that whereas IS25 is an agonist of TRβ with an EC_50_ of 448 nM [29], TG68 (its acetamide analog) has been designed as a pro-drug of IS25 with improved drug-like properties and bioavailability over the zwitterion IS25 [29]. Recent investigations conducted on cellular and animal models of pathological conditions including non-alcoholic steatohepatitis (NASH) and hepatocarcinoma (HCC), characterized by a defect of the T3 pathway, have confirmed the comparable activity of both compounds. More importantly, TG68 and IS25 both stimulate hepatocyte proliferation without T3/TRα-dependent side effects [32] and promote lipolysis in HepG2 cell lines, a mechanism mediated at least in part by AMP-activated protein kinase (AMPK).

The different efficacy profiles of TG68 and IS25 in OPC differentiation suggest a different modulation of the receptor or possibly a different mode of action. Other thyromimetic drugs such as CGS23425, DITPA, KB2115, and the HepDirect pro-drug MB07811 have been proposed to mimic the molecular action of T3 and tested for their ability to induce the downstream effects mediated by TRs, notably in the setting of OPC differentiation [49]. When comparing the action of these thyromimetics to T3, however, differences emerge based on the cell type and the considered readout. While due in part to the contrasting expression of TRs in the various systems and the different TR selectivity of the tested ligands, these differences are mostly due to unknown molecular reasons [50].

T3-mediated genomic action is in fact a complex orchestration of conformational TR changes that modify their affinity for different co-regulators, controlling the expression of the target genes. These mechanisms appear to be cell- and gene-specific since T3 acts either as an inducer or a repressor, depending on the considered target and cell type. The underlying molecular details, however, along with the entire list of cell-specific T3 target genes, have yet to be identified [13]. Other points to consider are that the complex differentiation machinery involves a number of other players, including extracellular matrix components, growth factors, and cell signals, and that thyromimetics may act on other unexpected targets.

Although it is not yet known whether TRβ1 activation is sufficient to promote oligodendrogenesis [28], OL differentiation occurs through a number of distinct pathways, including AMPK modulation. Preliminary investigations have revealed the polypharmacology of both compounds tested in this study and, most importantly, the significant activity of TG68 on AMPK activation. AMPK is a serine/threonine kinase that serves as the primary gatekeeper of metabolic processes and plays a critical role in cell growth and survival. Mounting evidence indicates that it plays a key role in neurodegenerative disease and may also be involved in brain development, neuronal polarization, and neuronal activity. AMPK activation increases autophagic clearance of Aβ and tau aggregates, reduces oxidative stress and tau phosphorylation [51], and stimulates the myelination process in MS animal models [52].

Further research is needed to define the mechanism by which the pleiotropic effects of TG68 support oligodendrocyte differentiation and myelin regulation and to confirm whether AMPK stimulation contributes to the effects driving OPC differentiation.

### 3.2. Implications for CNS Lesion Repair and Neurodegenerative Diseases

While the use of THs and their analogs for therapeutic purposes has been the focus of increasing interest over the past few decades [53,54,55], most thyromimetic drugs proposed for the promotion of OPC differentiation in demyelinating diseases have not passed clinical trials, such as GC-1, which was stopped at phase one, and KB2115 (Eprotirome), terminated at phases 2–3 [32,56]. To date, only one short-term phase 1 study has been performed on MS patients (NCT02760056, www.clinicaltrials.gov; accessed on 7 April 2023) [57] out of the numerous synthetic TRβ ligands developed [58]. The novel compound TG68 has the remarkable property of promoting OPC differentiation, even in the presence of inflammatory challenges. We previously showed that the sustained inflammatory microenvironment associated with inflammatory/demyelinating diseases leads to a local dysregulation of T3 metabolism, directly mediated by the inflammatory cytokines, which increase the expression of DIO3, the T3-inactivating enzyme, and down-regulate the TRs, thus blocking the activation of the differentiation machinery [26,27,33]. The DIO3 activity is exerted by the removal of the iodine substituent in the five position from the tyrosyl ring of T3 or T4, generating the inactive forms of the hormone, T2 and rT3, respectively [59]. The synthetic ligand TG68, like the other principal thyromimetics, is not a substrate of the deiodinase enzymes, a structural characteristic that makes it a putative therapeutic agent for overcoming the inflammation-mediated OPC differentiation block.

## 4. Materials and Methods

### 4.1. Cell Cultures and Treatments

To obtain an OPC-enriched culture, fetal NSCs (from E13.5 forebrains) were isolated according to previously published protocols [33]. In brief, fetal heads were positioned in a Petri dish containing PBS 1 × with 1% Penicillin/Streptomycin (P/S; Thermo Scientific, Waltham, MA, USA) (100 U × mL^−1^/100 µg × mL^−1^). Under a dissection microscope, the brains were removed using open forceps and placed upright on the dish. The meninges were carefully detached and removed using forceps, and the forebrains were collected in a 1.5 mL tube. The PBS was removed, and the tissues were incubated with non-enzymatic dissociation buffer. After 15 min incubation at 37 °C, the tissues were mechanically dissociated by pipetting several times. After 5 min of centrifugation at 400× *g*, the cellular pellet was resuspended in serum-free NSC medium (DMEM/F12 GlutaMAX; 8 mmol/L HEPES; 100 U/100 μg/mL P/S; 1 × B27; 1 × N2; 10 ng/mL bFGF; 10 ng/mL EGF; (Thermo Scientific, Waltham, MA, USA), and the cells were plated at a density of 10 cells/µL in a T25 flask (Corning, New York, NY, USA) following cell count. The flask was held vertically to avoid cell attachment, allowing the establishment of cell suspension cultures, and the medium was changed every three days. Neurospheres were allowed to proliferate until they reached a diameter of about 100–150 µm, at which point the three-dimensional spheroid structures were centrifugated at 400× *g* for 5 min and mechanically dissociated by pipetting several times. To obtain the OPC-enriched spheres (oligospheres), the cells were counted and plated again at a density of 10 cells/µL in OPC medium (DMEM/F12 GlutaMAX; 8 mmol/L HEPES; 100 U/100 μg/mL Penicillin/Streptomycin; 1 × B27; 1 × N2; 20 ng/mL bFGF; 20 ng/mL PDGF; Thermo Fisher Scientific). When the oligospheres reached a diameter of approximately 100–150 µm once more, they were again centrifuged and dissociated in a single-cell suspension. Following cell count, the cells were plated at a density of 3000 cells/cm^2^ on pre-treated poly-D,L-ornithine (50 µg/mL)/laminin (5 µg/mL; Sigma-Aldrich) coated wells or coverslips, again in OPC medium.

After 3 DIVs, the OPC medium was replaced with the oligodendrocyte differentiation medium (DMEM/F12 GlutaMAX; 8 mmol/L HEPES; 100 U/100 μg/mL Penicillin/Streptomycin; 1 × B27; 1 × N2; 50 nM T3; 10 ng/mL CNTF; 1× *N*-acetyl-L-cysteine; Thermo Fisher Scientific) to induce OL differentiation and maturation.

The characterization of the cell culture composition has been included in the Supplementary Materials (Supplementary Figure S1).

Treatment with TR ligands was performed at the same time as differentiation induction by physiological T3 exposure. In the first set of experiments, we tested three doses of IS25 and TG68 (1, 10, and 50 µM) to determine the optimal dose. Cultures were also treated with vehicle (DMSO 0.001%) and exposed to physiological T3 differentiation induction (50 nM) as a control group. The cultures were analyzed for cell culture composition in terms of OPC differentiation at 6 DIVs (Figure 1A).

In the second set of experiments, we treated the cultures with a single dose of the two molecules, the positive T3 control and the synthetic TRβ ligand GC-1, analyzing the entire maturation process of the OL population (Figure 1D).

The selected dosage was also used in all the other experiments to analyze OL maturation (Figure 2A), gene expression regulation (Figure 2D), and the ability of the selected compound to overcome the cytokine-mediated OPC differentiation block (Figure 3A).

### 4.2. Cytokine Mix Exposure

Following a well-established protocol [33], OPC-enriched spheres were exposed during the proliferation phase to a cytokine mix consisting of 10 ng/mL each of IL-1β (OriGene, cat no. TP723210), IL-6 (OriGene, cat no. TP723240), TNFα (OriGene, cat no. TP723451), TGFβ (OriGene, cat no. TP300973), IL17 (OriGene, cat no. TP762309), and IFNγ (OriGene, cat no. TP723744). The control cultures were treated with the cytokine mix vehicle (0.8% of solution consisting of 10% glycerol, 100 mM glycine, and 25 mM Tris at pH 7.3). Exposure lasted for the entire oligosphere generation period of 7 days.

Cells were then seeded in a 96-well plate, treated with physiological T3 exposure (50 nM) or TG68 (10 µM) and cultured until 12 DIVs.

### 4.3. RNA Extraction and Gene Expression Analysis

Cells were seeded in a 24-well plate and lysed after 1, 3, and 6 DIVs following treatment with vehicle (DMSO 0.01%), T3, or TG68.

Total RNA was extracted using the RNeasy micro kit (Qiagen) and eluted in RNase-free water. Absorbance values at 260, 280, and 320 nm were then measured using a spectrophotometer (Nanodrop 2000, Thermo Scientific).

The same amount of starting RNA was used for the reverse transcription reaction (500 ng), using the iScript™ cDNA Synthesis kit (Bio-Rad). A no-RT control containing the entire reaction mix without the enzyme was produced in parallel with all the other samples to check for possible genomic DNA contamination.

Semi-quantitative real-time PCR was performed using the CFX96 real-time PCR system (Bio-Rad, Hercules, CA, USA). The reactions were performed in a final volume of 20 µL consisting of SYBR Green qPCR master mix (Bio-Rad), 0.4 µM forward and reverse primers, and nuclease-free water. The no-RT control was processed in parallel with the others and tested for every primer pair using real-time PCR. No-template controls were also added for each gene expression analysis. The primers were designed using Primer Blast software (NCBI, Bethesda, MD, USA) and synthesized by IDT (Coralville, IA, USA): *Klf9*, FW: 5′-AGTGGCTTCGAAGGGGAAAC-3′, REV: 5′-TCCGAGCGCGAGAACTTTTT-3′; *Gapdh*, FW: 5′-GGCAAGTTCAATGGCACAGTCAAG-3′, REV: 5′-ACATACTCAGCACCAGCATCACC-3′.

The thermal profile of the PCR reactions consisted of one denaturation step (98 °C, 3 min) and 40 cycles of amplification (95 °C for 10 s, 60 °C for 1 min). At the end of the amplification cycles, the dissociation curve was obtained by first incubating samples at 95 °C for 1 min to denature the PCR-amplified products, followed by ramping the temperature down to 65 °C, before finally increasing the temperature from 65 °C to 95 °C at a rate of 0.5 °C/s, continuously recording the fluorescence intensity throughout the temperature ramp.

For the gene expression analysis, we used the 2^−(ΔΔCt)^ method, initially normalizing the gene of interest on the housekeeping gene, before expressing the results as a relative fold-of-change compared to the vehicle control group at each considered time point.

### 4.4. Immunocytochemistry and Image Analysis

Indirect immunofluorescence procedures were used to identify OPCs (NG2) and mature OLs at early (CNPase) and late (MBP) stages.

Cells were washed in PBS and fixed in 4% paraformaldehyde in 0.1 M Sørensen phosphate buffer for 20 min at room temperature. After rinsing 2 times in PBS, cells were blocked with 1% Donkey Normal Serum (Sigma-Aldrich, Burlington, MA, USA) in PBS for 1 h at room temperature, then incubated overnight at 4 °C in a humid atmosphere with the primary antibody diluted in 0.3% PBS/Triton-X100 (Merck-Millipore, Burlington, MA, USA): anti-NG2 (Merck-Millipore, Burlington, MA, USA; rabbit, 1:250); anti-CNPase (Merck-Millipore, Burlington, MA, USA; mouse, 1:250); anti-MBP (Dako, Glostrup, Denmark; rabbit, 1:250); and anti-GFAP (Dako, Glostrup, Denmark rabbit; 1:1000). After two washes in PBS, samples were incubated with the secondary antibodies for 30 min at 37 °C: Alexa fluor 568-conjugated anti-mouse (molecular probes) and Alexa fluor 488-conjugated anti-rabbit (Invitrogen, Waltham, MA, USA). The Hoechst 33,258 nuclear dye (Invitrogen, Waltham, MA, USA, cat no. H3569) was added at a concentration of 1 μg/mL during incubation of the secondary antibodies.

The cells cultured in the 96-well plate were analyzed using cell-based high content screening (HCS) technology (Cell Insight XT; Thermo Scientific, Waltham, MA, USA). The dedicated software (HCS Studio v 6.6.0) was used for acquisition and analysis, selecting the “cell morphology” algorithm, which detects the whole cell population using nuclear staining and the cell body based on cytoplasmic fluorescence intensity. Setting the intensity threshold for each specific marker, the HCS machine automatically analyzes the entire experimental set and quantifies the percentage of each cell type through a protocol set up in detail by our group [33].

Images from cells cultured on coverslips in 24-well plates were acquired by an epifluorescence microscope (Nikon Eclipse E600, Nikon, Tokyo, Japan, equipped with a Q Imaging Retiga-2000RV CCD camera) and Nis-Elements AR 4.3 software. To analyze the complexity of the mature MBP-positive OL membrane, we used the Fiji software (ImageJ v.2.0.0-rc-69/1.52p) and the dedicated plug-in to perform the Sholl analysis.

### 4.5. Statistical Analysis

Data are reported as mean ± SEM. Three to five independent replicates were used for each experimental set. For the differentiation analysis performed by HCS, the entire cell population in each well was analyzed (20,000–30,000 cells/well), increasing the statistical power of the cell platform and avoiding the operator-dependent bias inherent in randomly choosing representative fields per well.

Prism software (GraphPad PRISM v 8.0.1) was used for statistical analyses and graph generation. Statistical analysis was based on one-way ANOVA and Dunnett’s or Tukey’s post-hoc test for comparison between more than two groups. Results were considered significant when the probability of their occurrence due to chance alone was less than 5% (*p* < 0.05).

## 5. Conclusions

In conclusion, the presented evidence produced using robust in vitro models paves the way for further studies on the therapeutic potential of TG68 and the TR-dependent and independent mechanisms involved in OPC differentiation.

## Data Availability

Data is contained within the article and Supplementary Materials.

## Supplementary Materials

The following supporting information can be downloaded at: https://www.mdpi.com/article/10.3390/ph16091207/s1, Figure S1: Characterization of the NSC-derived OPC cultures. (A) Cells were isolated from the forebrain of mouse embryos (E13.5) and cultured in suspension as neurospheres adding in the culture medium EGF and bFGF. Neurospheres were splitted and replated in suspension adding in the medium PDGF-AA and bFGF to obtain a first driving through the oligodendroglial lineage, obtaining the oligospheres. Spheres were dissociated and plated in adhesion as on Poly-D-ornithine/laminin coating in the same culture medium to obtain an OPC-enriched culture (-3 DIV). After 3 days, the culture medium was replaced with the differentiation medium, deprived of the growth factors and containing T3 to induce the oligodendrocyte differentiation/maturation (DIV 0). (B) Representative images of spheres, astrocytes (GFAP-positive cells), OPCs (NG2-positive cells), and mature oligodendrocytes (CNPase/MBP-positive cells), are included in the figure. Bars: 150 µm; 100 µm. C) Graph shows the culture composition before the T3-drive differentiation induction (DIV 0) and at the end of the considered differentiation/maturation period (DIV 12). Quantifications of astrocytes (GFAP-positive cells), OPCs (NG2-positive cells), and pre-oligodendrocytes (CNPase-positive cells) and mature-oligodendrocytes (MBP-positive cells) have been included. Figure S2: HCS-derived images Figure 1. Figure shows representative images of cell-based High Content Screening-derived pictures acquired at 10× for the analysis included in the main text, Figure 1, of cultures exposed to T3, GC1, IS25 or TG68 after 12 DIVs of treatment, stained to detect OPCs (NG2) and late-mature OLs (MBP). Epifluorescence images of MBP-positive cells are included for each group. Bars: 150 µm. Figure S3: Analysis of the astrocyte lineage. (A) Graph shows the quantification of the GFAP-positive population (astrocytes). (B) Representative images of the experimental groups are included. Statistical analysis. Bars represent mean + SEM; n = 3 independent replicates. One-way ANOVA followed by Tukey’s post-test. Asterisks represent differences between the indicated groups and the T3 control group (* *p* < 0.05; ** *p* < 0.01). Figure S4: HCS-derived images Figure 3. Figure shows representative images of cell-based High Content Screening-derived pictures acquired at 10× for the analysis included in the main text (Figure 3) of cultures exposed and non-exposed to cytokines and treated with T3 or TG68 after 12 DIVs, stained to detect early mature OLs (CNPase) and late-mature OLs (MBP). Bar: 150 µm.
