# Supplementary material for: Synthetic Thyroid Hormone Receptor-β Agonists Promote Oligodendrocyte Precursor Cell Differentiation in the Presence of Inflammatory Challenges
Source: Pharmaceuticals (Basel). 2023 Aug 25;16(9):1207. doi: 10.3390/ph16091207 (PMC10534456; doi:10.3390/ph16091207)
Supplement: Supplementary file 1 [file pharmaceuticals-16-01207-s001.zip › pharmaceuticals-2531445-supplementary.pdf]

SUPPLEMENTARY MATERIALS

Article

Synthetic Thyroid Hormone Receptor-β Agonists Promote Oligodendrocyte Precursor Cell Differentiation in the Presence of Inflammatory Challenges

Vito Antonio Baldassarro [1], Corinne Quadalti [2], Massimiliano Runfola [3,†], Clementina Manera [3], Simona Rapposelli [3] and Laura Calzà [2,4,*]

[1] Department of Veterinary Medical Science (DIMEVET), University of Bologna, 40064 Bologna, Italy; vito.baldassarro2@unibo.it
[2] Department of Pharmacy and Biotechnology (FaBit), University of Bologna, 40126 Bologna, Italy; corinne.quadalti2@unibo.it
[3] Department of Pharmacy, University of Pisa, 56126 Pisa, Italy; massimiliano.runfola@pharm.ox.ac.uk (M.R.); clementina.manera@unipi.it (C.M.); simona.rapposelli@unipi.it (S.R.)
[4] IRET Foundation, Ozzano Emilia, 40064 Bologna, Italy
* Correspondence: laura.calza@unibo.it
† Current address: Department of Pharmacology, University of Oxford, Oxford OX1 4BH, UK.

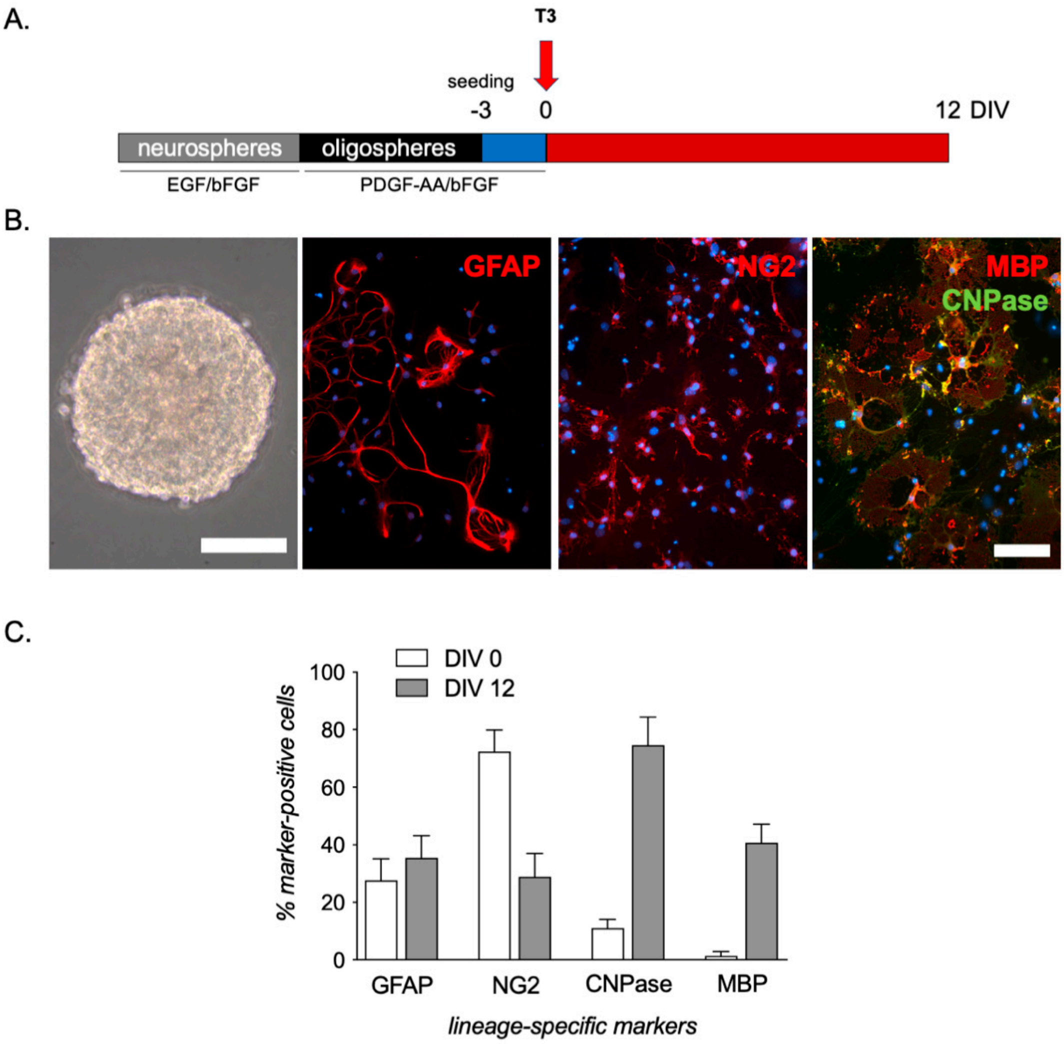

Figure S1. Characterization of the NSC-derived OPC cultures.

(A) Cells were isolated from the forebrain of mouse embryos (E13.5) and cultured in suspension as neurospheres adding in the culture medium EGF and bFGF. Neurospheres were splitted and replated in suspension adding in the medium PDGF-AA and bFGF to obtain a first driving through the oligodendroglial lineage, obtaining the oligospheres. Spheres were dissociated and plated in adhesion as on Poly-D-ornithine/laminin coating in the same culture medium to obtain an OPC-enriched culture (-3 DIV). After 3 days, the culture medium was replaced with the differentiation medium, deprived of the growth factors and containing T3 to induce the oligodendrocyte differentiation/maturation (DIV 0).

(B) Representative images of spheres, astrocytes (GFAP-positive cells), OPCs (NG2-positive cells), and mature oligodendrocytes (CNPase/MBP-positive cells), are included in the figure. Bars: 150 µm; 100 µm.

(C) Graph shows the culture composition before the T3-drive differentiation induction (DIV 0) and at the end of the considered differentiation/maturation period (DIV 12). Quantifications of astrocytes (GFAP-positive cells), OPCs (NG2-positive cells), and pre-oligodendrocytes (CNPase-positive cells) and mature-oligodendrocytes (MBP-positive cells) have been included.

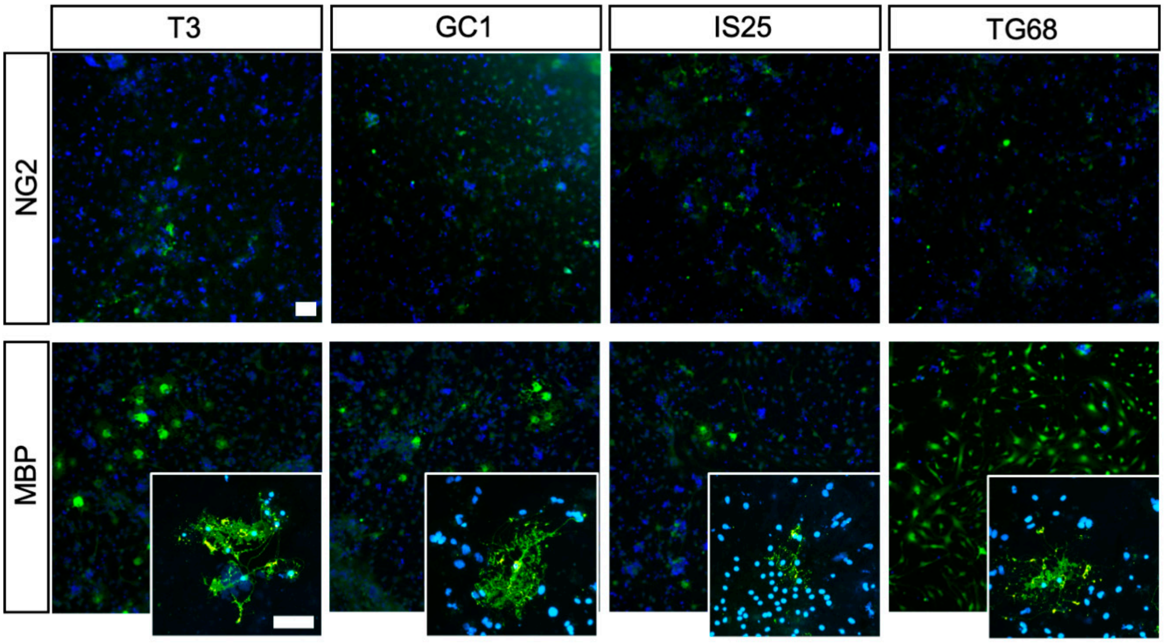

Figure S2. HCS-derived images Figure 1.
Figure shows representative images of cell-based High Content Screening-derived pictures acquired at 10× for the analysis included in the main text, Figure 1, of cultures exposed to T3, GC1, IS25 or TG68 after 12 DIVs of treatment, stained to detect OPCs (NG2) and late-mature OLs (MBP). Epifluorescence images of MBP-positive cells are included for each group. Bars: 150 μm.

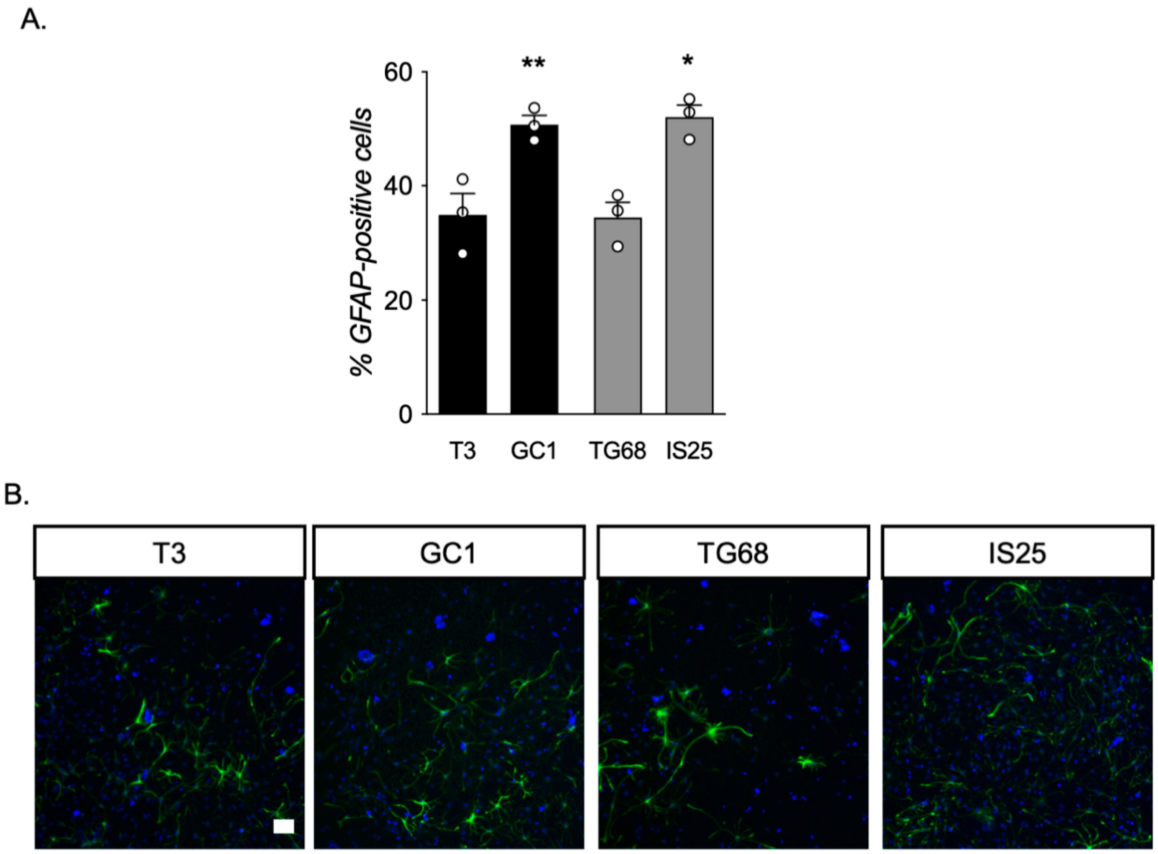

Figure S3. Analysis of the astrocyte lineage.
(A) Graph shows the quantification of the GFAP-positive population (astrocytes). (B) Representative images of the experimental groups are included. Statistical analysis. Bars represent mean + SEM; n = 3 independent replicates. One-way ANOVA followed by Tukey's post-test. Asterisks represent differences between the indicated groups and the T3 control group (* $p < 0.05$; ** $p < 0.01$).

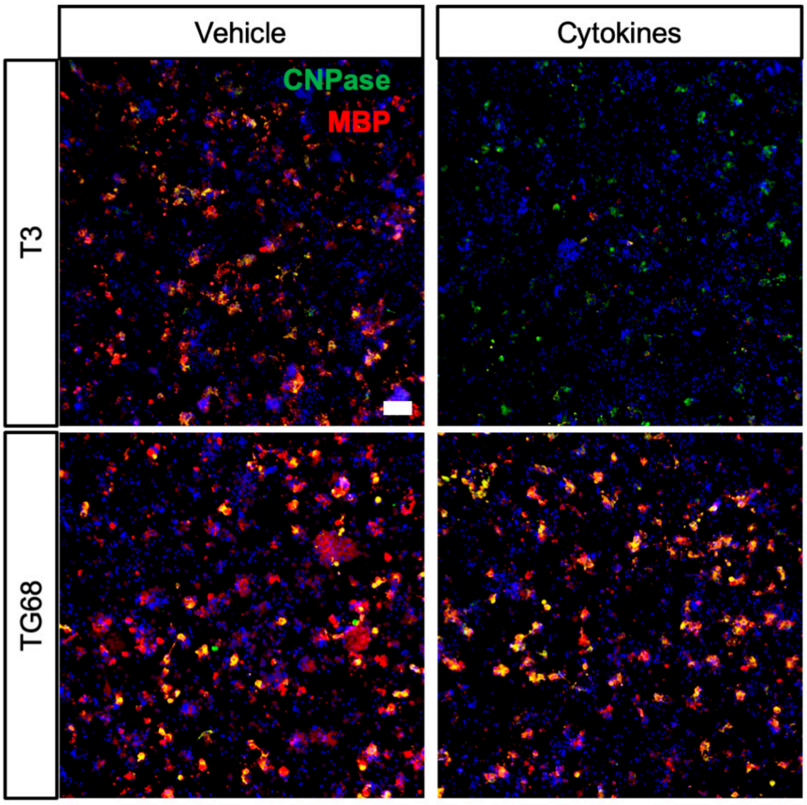

Figure S4. HCS-derived images Figure 3.
Figure shows representative images of cell-based High Content Screening-derived pictures acquired at 10× for the analysis included in the main text (Figure 3) of cultures exposed and non-exposed to cytokines and treated with T3 or TG68 after 12 DIVs, stained to detect early mature OLs (CNPase) and late-mature OLs (MBP). Bar: 150 μm.
